# Don’t you tweet me badly: Anxiety contagion between leaders and followers in computer-mediated communication during COVID-19

**DOI:** 10.1371/journal.pone.0264444

**Published:** 2022-03-04

**Authors:** Dritjon Gruda, Adegboyega Ojo, Alexandros Psychogios

**Affiliations:** 1 School of Business, National University of Ireland Maynooth, Maynooth, Ireland; 2 Birmingham City Business School, Birmingham City University, Birmingham, United Kingdom; 3 ALBA Graduate Business School, The American College of Greece, Athens, Greece; West Pomeranian University of Technology, POLAND

## Abstract

Do organizational leaders’ tweets influence their employees’ anxiety? And if so, have employees become more susceptible to their leader’s social media communications during the COVID-19 pandemic? Based on emotional contagion and using machine learning algorithms to track anxiety and personality traits of 197 leaders and 958 followers across 79 organizations over 316 days, we find that during the pandemic leaders’ tweets do influence follower state anxiety. In addition, followers of trait anxious leaders seem somewhat protected by sudden spikes in leader state anxiety, while followers of less trait anxious leaders are most affected by increased leader state anxiety. Multi-day lagged regressions showcase that this effect is stronger post-onset of the COVID-19 pandemic compared to the pre-pandemic crisis context.

## Worrying me softly with your tweets: Anxiety contagion between leaders and followers in computer-mediated communication during COVID-19

Emotional contagion, “a process in which a person or group influences the emotions or behavior of another person or group through the conscious or unconscious induction of emotion states and behavioral attitudes” [1, p. 50], plays an important role in several domains, including social interactions and leadership [2]. Previous work has identified that in face-to-face leader-follower interactions, leaders do not only transfer emotions to their followers via tacit, subconscious processes, but also can transfer emotions deliberately “with the intention of attaining certain interactions or task outcomes” [3, p. 659]. Leaders are likely to influence follower affect, due to leaders’ status role, which corresponds to access to resources and authority to shape the work environment as leaders see fit [4]. However, less is known about the transfer of emotions between organizational leaders and followers in computer-mediated communication (CMC) on public social media platforms such as Twitter. The presented research addresses this gap.

Based on emotional contagion, we explore the relationship between leader and follower anxiety. We focus on anxiety due to its state and trait properties [5]. And while previous studies have focused on the dispositional differences and situational characteristics that trigger anxiety [6], a longitudinal examination of the transfer of leader anxiety to followers, in particular using CMC, has not been conducted. In this respect, our study responds to the need of understanding further emotional contagion in organizational life [7] by examining a widely used and powerful means of communication between leaders and followers. Finally, we explore possible changes in the transfer of anxiety from leaders to followers before and during a threatening situation, namely the COVID-19 pandemic. Anxiety is considered the default crisis emotion [8], given that individuals in a crisis (i.e., a particularly threatening) situation experience anxiety more intensely and frequently than any other crisis emotion [incl. anger, fear or sadness; 8]. Hence, we examine whether during a crisis the occurrence and impact of anxiety contagion from leaders to followers differ compared to normal circumstances. We examine this particular phenomenon in an exploratory manner, using an innovative ML approach to detect both state and trait anxiety [9] in a large sample of 197 leaders and 958 followers and derive 43,283 daily indications of anxiety from posts and interactions on the Twitter platform by leaders and followers over 316 days.

### Emotional contagion in face-to-face and computer-mediated communication

Emotional contagion constitutes the transfer and sharing of emotions from one person to another [7]. As stated by Barsade, Coutifaris [7], emotional contagion is 1) comprised of distinct emotions (e.g., anger, anxiety), 2) “occurs via subconscious and conscious processes that transpire when people are both elicitors and targets of emotional contagion” [(p. 138), 3] occurs on an interpersonal level and finally 4) not only influences “how people feel but also what they subsequently think and do” [p. 138].

Based on this definition, we examine emotional contagion as a social influence, in which “[person] A has power over [person] B to the extent that [A] can get B to do something that B would otherwise not do” [10, pp. 202–203], while the power of person A over person B is affective in nature. If person A is successful in transferring their emotions to person B, person B non-consciously imitates the communicated affect, which leads to convergence in both interaction partners’ emotions [11]. Importantly, previous research oftentimes equates emotions with affect–a term that comprises all aspects of subjective feelings [12]. To stay consistent with past research and avoid confusion, throughout this manuscript, we use the term emotional contagion when describing the transfer of anxiety, as part of follower affect. Similar to previous studies (Barsade, 2018), we do distinguish between emotions, short-term affective reactions to stimuli, and dispositional affect, a trait-like predisposition to experience certain feelings at any given time.

The majority of scholars have studied the occurrence and underpinnings of emotional contagion in face-to-face interactions [for a review see 7]. However, there is some indication that emotional contagion is not only possible in CMC but also that emotions effectively can spread both directly and indirectly in social networks such as social media. For example, Cheshin, Rafaeli [2] confirmed that the transfer of emotion also occurs in virtual teams and that these “text-based communications of emotion were detected and ‘caught’ by partners interacting via text-based instant messaging” (p. 3)]. And in dyadic CMC interactions partners exchanged messages slower and used shorter messages when experiencing negative emotions, compared to participants experiencing neutral emotions [11]. Interaction partners in CMC could also distinguish discrete emotions in messages, namely anger or happiness. Hence, it seems clear that when communicating electronically individuals may influence others affectively [7] and even impact behavioral outcomes [3].

Yet, the majority of existing studies have examined emotional contagion in CMC using text-based communication on Web 1.0 platforms, i.e. communication between two individuals via text, largely ignoring another mainstream means of communication–social media. Barsade, Coutifaris [7] point out, social media platforms are much more open, interactive, and dynamic, allowing communication between an individual and entire groups or communities at once. Hence, social media platforms allow researchers to study the effects of emotion transfer from leader to several followers (i.e., from A→B and A→C) simultaneously [13]. Indeed, previous studies have found that emotions can spread between users via CMC on social media platforms [14], and can do so from one user to several others [11, 15]. Yet, less is known about the spread of discrete emotions via CMC, in particular, whether some emotions are more likely to be spread and transferred to others [7]. And as the use of public social media platforms is becoming more prevalent and accepted by both organizations and employees [16], we argue that more attention needs to be paid to the study of emotional contagion from leaders to followers, and its repercussions on follower affect, via CMC.

### Anxiety contagion between leaders and followers

Anxiety is characterized by negative valence and high arousal, as well as cognitive appraisals of uncertainty and low control [17, 18] and is a transient emotional response to an event- or situation-specific context. Throughout this paper anxiety is based on the presented sub-clinical definition, as opposed to the clinical diagnosis of anxiety. This transient episode of anxiety, triggered by a threatening or uncertain situation, is referred to as state anxiety. State anxiety is short in duration, unlike mood or other forms of dispositional affect [19], and is particularly important in the transfer of emotion in communication because anxiety is functional and “can facilitate constructive behavior” [19]. For example, in response to situation-specific threats, anxious individuals are more likely to detect and recognize potential threats, protect themselves or others, and in general, become more vigilant [20]. Hence, anxiety can help individuals adapt to potentially threatening and harmful environmental demands, especially when facing overwhelming threats such as a crisis. However, most previous research has not focused on the transfer of anxiety but rather negative and positive affect in general.

In addition, to the best of our knowledge, no previous studies have examined anxiety contagion between organizational leaders and followers using social media. Yet, based on previous evidence of emotional contagion between users of social networks [11, 21] and the signaling power that organizational leaders possess, we would expect leader state anxiety via CMC to influence follower state anxiety over time.

*Hypothesis 1*: *In CMC*, *leader state anxiety positively predicts follower state anxiety*.

### The role of trait anxiety

It is important to note that the predisposition to anxiety can vary between individuals in regards to both intensity and frequency. Hence, some individuals have a greater tendency to experience anxiety [5], known as trait anxiety. Trait anxiety, in combination with state anxiety, influences an individual’s magnitude of experienced anxiety in any given situation [19]. Individuals who are predisposed to experience anxiety (i.e., trait anxiety) tend to perceive situations as more threatening, pay more attention to presented negative information, and tend to experience stronger physiological and psychological sensations [22]. Therefore, trait anxiety plays a significant role in determining the intensity and frequency of experienced state anxiety.

Concerning the leader-follower relationship, we know that leader trait affectivity can influence contagion susceptibility but also can “influence leaders’ ability to influence others through emotion” [3, p. 658].

Hence, we would expect followers of high trait anxiety leaders to be more used to their leader displaying heightened anxiety to situations in general. Hence, it is likely that when leaders express heightened state anxiety, followers of trait anxious leaders are likely to express less state anxiety compared to followers of leaders who rarely exhibit anxiety. We hypothesize the following:

*Hypothesis 2*: *In CMC*, *leader trait anxiety moderates the relationship between leader state anxiety and follower state anxiety*, *in that followers of highly trait anxious leaders experience less increased state anxiety than followers of low trait anxious leaders*.

### The role of context

While more anxious leadership could be associated with more anxious followers in general, this might change due to context, such as a crisis as “individuals differ in their adaptation to events, with some individuals changing their set point and others not changing in reaction to some external event” [23, p. 306]. Hence, we argue that the transfer of anxiety from organizational leaders (i.e., individuals with resources and authority) to their followers would be particularly useful to examine considering the critical role anxiety plays in crises. Accordingly, we examine the relationship between leader and follower anxiety in pre-and during crisis contexts, namely the COVID-19 pandemic.

#### Emotional contagion during the COVID-19 pandemic

With terms such as “social distancing” and “flattening the curve” becoming part of everyday vocabulary, the COVID-19 pandemic is the most challenging health crisis of the 21^st^ century [24]. Coronavirus officially was declared a worldwide pandemic by 11^th^ March 2020 by the World Health Organization, with governmental work-from-home orders following shortly thereafter. Employees soon found themselves in a new and, for most, quite unfamiliar social territory, with homes all over the world converting into offices, schools, daycares, gyms, etc. while communication to the outside world mainly moved online. The changing working conditions and the increased economic pressures have led to a wider socioeconomic crisis, typically followed by adverse working conditions for employees [25]. Given the increased need for remote working, managers and employees alike have turned to CMC as a primary means of communication.

Yet, research on information communication and sharing in a crisis (or crisis-like situation) is limited. One study examined how communication on Twitter occurs during the Great East Japan Earthquake in 2011 [26] and concurs with findings of related studies that negative feelings, such as worry and anxiety, were more likely to be shared online than neutral or positive feelings [27]. As information grows explosively in a crisis, people flock online, especially to public communication platforms such as Twitter, to communicate and share information with others [28]. Individuals also rely on these platforms to discover and evaluate how others have been affected by a crisis and in turn can use these platforms to emotionally influence others [29]. In sum, via social media individuals are both influenced by and “influence each other more instantly and frequently” [27, p. 2033]. Therefore, communication on social media platforms provides a feasible alternative to study emotional contagion between leaders and followers, especially in the case of a crisis marked by social distancing and a strong reliance on CMC between organizational members.

Nevertheless, it is important to remember that not all crises are the same [30], nor do all crises impact all individuals to the same degree [24]. For example, Barsade, Coutifaris [7] suggest that in the last financial crisis, individuals who were in financial distress were more likely influenced by the anxiety described in the press and social media, and making the transferred anxiety their own, which in turn affected behavioral outcomes such as restricted spending. However, in the case of the COVID-19 pandemic, the affective impact of the crisis itself is not yet clearly defined. On the one hand, previous studies have found that state anxiety levels have increased for entire populations [31], on the other hand, recent studies have also found that exogenous individual differences such as age, risk behavior, and resilience also influence state anxiety [24].

In short, the COVID-19 crisis provides a unique setting, in which interactions between leaders and followers may be studied in extreme circumstances, and compared to CMC in normal circumstances (i.e., before the onset of the COVID-19 pandemic). Yet, due to the uniqueness of the COVID-19 crisis concerning its (universal vs. individualized) influence on employees’ affect, we hesitate to form specific hypotheses regarding pre-and-during-crisis differences in the relationship between leader and follower anxiety. Hence, we decided to examine the COVID-19 pandemic context as a possible moderating factor in an exploratory manner.

## Methodology

Due to the state- and trait-like properties of anxiety, it is crucial to study follower anxiety over time [9]. The presented study overcomes typical diary study limitations (e.g., high dropout rates of respondents) by applying anxiety and personality detection algorithms on a large sample of leaders and followers. The final sample comprised 197 leaders and 958 followers in 79 companies engaging in CMC on the public social media platform Twitter. This resulted in a total of 43,283 matched daily leader-follower observations. The respective minimal anonymized data can be found on Open Science Framework (https://osf.io/3r8z7/). The collection method complied with the terms and conditions for the websites from which the data was collected.

### Sample and procedure

Our methodology comprises five key steps. The first step involves the preparation and pre-processing of our dataset. Using an initial database of organizational leaders and employees (https://crunchbase.com), and their social media information, we selected organizations and respective employee social media data based on the number of listed social media handles. An organization was included as long as it listed at least ten employees and their public social media information in our dataset. We define leaders as C-suite executives, i.e., individuals with a job title of e.g., Chief Executive Officer (or CEO), Chief Financial Officer (or CFO), etc. The remaining individuals were classified as followers. Notably, we only included employees from the United States of America, as the anxiety detection algorithm was trained exclusively on U.S. data (see section “Predicting state and trait anxiety”). We recognize that this categorization approach is limited, primarily for two reasons. Firstly, this distinction between leaders and followers does not allow us to match followers with their direct supervisors. However, C-suite executives are largely recognized as senior organizational leaders [4], and therefore wield greater signaling power, which in turn influences all employees [32]. Secondly, one could argue that high-level leaders might be inclined to censor their social media communications to ensure that a positive message is conveyed. Such communications also might be crafted with assistance from the organization’s communications team or even HR since they enhance employee voice [33]. While we agree with this argument, we would add that because emotional contagion can occur both purposefully or intendedly [34], leaders’ communications still affect their followers even if leaders are not themselves tweeting. In other words, emotional contagion still occurs independently whether emotions are communicated on purpose or unintentionally.

In addition, to further ensure the validity and accuracy of provided information of leaders and followers and increase the robustness of the provided data, we manually searched all included leaders and followers on the LinkedIn platform (https://linkedin.com). Doing so allowed us to confirm that leaders and their respective followers were indeed employed in their respective organizations throughout the examined timeframe, including pre-and post-onset of the COVID-19 pandemic.

Finally, we chose to include gender as an additional control in our model, because gender is an exogenous variable that cannot be influenced by the outcome variable. Due to the limitations of the Twitter API (twitteR Package documentation - https://rpubs.com/Kyleen1991/594933), a maximum of 3,200 tweets per profile were extracted.

#### Predicting state and trait anxiety

In the second step, we annotate the dataset with an anxiety prediction algorithm. Extracted tweets of all available leaders and followers curated in the previous step were annotated using the anxiety detection algorithm as described in Gruda and Hasan [9]. This algorithm was trained on a dataset of 600 randomly selected tweets from 10,386 users, scored by 604 zero-acquaintance human raters from the United States based on a six-item short-form of the Spielberger State-Trait Anxiety Inventory [STAI; 35]. On average, each tweet was rated five times. Each tweet was assigned an anxiety score of between 1 (“Not at all”; low anxiety) and 4 (“Very much”; high anxiety) by each rater. Two types of features were extracted from the texts of the tweets. The first type of feature is based on the pre-trained Global Vectors for Word Representation (Glove) embedding. The second type of feature comprises the unigram and bigram terms (including emojis) and the corresponding term frequency (TF). The ML algorithm was implemented as two Linear Ridge Regression models corresponding to the sets of features described earlier. For the prediction of anxiety scores of non-labeled tweets, the average of the two predicted scores from the two models is taken as the final score. The training procedure involved the use of a 6-fold cross-validation resampling plan and resulted in a model with R^2^ = 0.49 and a Root-Mean-Square Error (RMSE) of 0.52. The trained model was validated using a set of 3.33 million tweets. The model predicted the anxiety scores of the tweets to be between 1 and 4 for 99.7% of the tweets (*M* = 2.34, *SD* = 0.36).

Trait anxiety was accounted for using the same algorithm as described above. Given that trait anxiety is traditionally measured as the frequency of anxiety experiences [9, 35], in this study trait anxiety constituted a 30 day average of anxiety scores per user, derived from available tweets before the examined period (before 5^th^ October 2019). This provided us with approximately one month of anxiety ratings per individual (i.e., leaders and followers) in our dataset.

Manual evaluation of the predicted anxiety scores for our dataset showed that the scores were highly correlated with the emotion expressed in the tweets. For instance, the tweet *“Thank you everyone—it’s been a busy week*! *We love our wonderful […] community*!*”* was predicted to have an anxiety score of 1.7217, while the tweet “*How it feels to be a web team fighting DDoS […]*” was assigned an anxiety score of 2.5432. Finally, the tweet *“[…] no no no*. *Not a happy place*.*”* was scored 3.099.

#### Predicting the Big-Five personality traits

Emotional contagion susceptibility may be influenced by individual differences. Hence, we measure and control for all Big Five leader and follower personality traits. Previous work [e.g., 36–39] has shown that personality traits can be measured accurately and successfully in online contexts using social media data. Therefore, in our third step, each Twitter profile was fed into the IBM Watson Personality Insights API, which extracts and analyzes social media textual data to identify personality traits based on linguistic analysis [40].

IBM Watson relies on an open-vocabulary machine-learning approach and is used to compute raw trait scores and subsequently compare raw scores to a reference sample of 1,000,000 individuals. A pre-condition for using this service is that the examined Twitter profiles need to be public and have a minimum of 100 words across tweets. The provided mean absolute error indicates the difference between estimated or predicted scores and actual scores. The IBM Watson Personality Insights algorithm provides a Watson estimates error rate of ca. 12%. In addition, the IBM Watson algorithm also provides six individual facets for each Big Five personality dimension [31, 40]. All facets were combined into higher-order Big Five personality dimensions (all Cronbach α ≥ .70, except for Openness to Experience: α = .68, see Table 1). Although a higher Cronbach alpha could have been achieved in the case of Openness to Experience by excluding the facet “adventureness” (α = .73), we decided to include all facets to ensure a full picture of the examined data.

**Table 1:** Pairwise correlations of main variables.

			M	SD	1	2	3	4	5	6	7	8	9
Leader	1	State Anxiety (Day 2)											
	2	State Anxiety (Day 1)	2.15	0.28	-	-							
	3	State Anxiety (Day 0)	2.15	0.28	-	0.19	-						
	4	Trait Anxiety	2.14	0.10	-	0.34	0.34	-					
	5	Openness to Experience	0.64	0.10	-	0.11	0.10	0.21	(.68)				
	6	Conscientiousness	0.62	0.14	-	-0.25	-0.25	-0.72	-0.18	(.86)			
	7	Extraversion	0.55	0.17	-	-0.27	-0.27	-0.75	-0.15	0.78	(.90)		
	8	Agreeableness	0.50	0.12	-	-0.19	-0.20	-0.58	0.31	0.76	0.74	(.73)	
	9	Neuroticism	0.33	0.19	-	0.24	0.24	0.71	0.17	-0.95	-0.83	-0.78	(.95)
	10	Gender	0.83	0.38	-	-0.01^†^	-0.01^†^	-0.01	-0.20	0.02^†^	-0.16	-0.09	0.02*
Follower	1	State Anxiety (Day 2)	2.21	0.28	-								
	2	State Anxiety (Day 1)	2.21	0.28	0.17	-							
	3	State Anxiety (Day 0)	2.21	0.28	0.17	0.17	-						
	4	Trait Anxiety	2.20	0.11	0.35	0.35	0.35	-					
	5	Openness to Experience	0.40	0.10	0.17	0.17	0.17	0.49	(.68)				
	6	Conscientiousness	0.54	0.14	-0.21	-0.21	-0.22	-0.63	-0.50	(.87)			
	7	Extraversion	0.42	0.16	-0.23	-0.24	-0.24	-0.68	-0.39	0.77	(.90)		
	8	Agreeableness	0.56	0.13	-0.16	-0.16	-0.16	-0.46	0.06	0.64	0.61	(.72)	
	9	Neuroticism	0.44	0.19	0.22	0.22	0.22	0.65	0.56	-0.93	-0.83	-0.60	(.95)
	10	Gender	0.74	0.44	0.04	0.04	0.04	0.10	-0.20	-0.04	-0.22	-0.27	0.04

*Note*: Cronbach-alphas in parentheses;, all other values are significant at *p* < .001, except values marked

* *p* < .05 or ^†^ (*p* > .05)

n = 197 leaders (n_observations_ = 11,446), 958 followers (n_observations_ = 45,708).

#### Post-annotation dataset consolidation

Our fourth step comprised the consolidation of state and trait anxiety scores (Step 2) with predicted Big-five personality traits (Step 3). From the resulting dataset, we formed pairwise combinations between leaders and followers, within companies, based on the time of the tweet. For example, tweets by followers from 1^st^ March 2020 were paired with leader tweets of the same day.

Due to the maximum number of (3,200) tweets limitations by the Twitter API, and since we also examine the role of context, specifically the COVID-19 crisis, as a secondary research question, we focused our main analyses on matched leader-follower observations between 5^th^ October 2019 and 13^th^ August 2020. Doing so provided us with a somewhat balanced dataset of 158 days before and 158 days after the onset of COVID-19. We classified the onset of COVID-19 to be the 11^th^ March 2020, during which COVID-19 was officially declared a worldwide pandemic by the World Health Organization.

Finally, since leaders and followers are nested within companies, we excluded companies with less than four daily leader-follower observations. Put differently, we disregarded cases in which there were less than four posted dyads between leaders and their respective followers on the same day. This threshold was identified as the bottom 10% of our dataset and was implemented to ensure data reliability on a company level. This final step resulted in a total of 43,283 matched daily leader-follower observations between 197 leaders and 958 followers across 79 companies.

#### Analytical strategy

In our dataset, followers are nested within leader-follower dyads, which in turn are nested within companies; observations in our dataset are not independent [41]. Hence, we use a multi-level cross-classified mixed-effects model for repeated measures to test our hypotheses. We define a three-level model with random intercepts at the company level. To compare the overall goodness of fit across models, we used the Aikake Information Criterion (AIC) and the Bayesian Information Criterion (BIC), which facilitate a comparison of mixed models with different numbers of levels and predictors. All analyses were conducted using Stata 16.0.

## Results

Summary statistics and pairwise correlations of variables on the leader and follower level are shown in Table 1. Results of our multi-level cross-classified mixed-effects model for repeated measures are shown in Table 2.

**Table 2:** Regression interaction between leader state anxiety (Day 1) and leader trait anxiety on subsequent follower state anxiety (Day 2).

	Model 1 (M1)		Model 2 (M2)		Model 3 (M3)	
Leader State Anxiety (Day 1)	0.01*	(2.37)	.22*	(2.21)	0.05	(0.41)
Leader State Anxiety (Day 0)	-0.00	(-0.05)	0.00	(0.27)	-0.00	(-0.00)
Follower State Anxiety (Day 1)	0.05***	(10.08)	0.05***	(9.81)	0.05***	(9.68)
Follower State Anxiety (Day 0)	0.04***	(9.19)	0.04***	(9.02)	0.04***	(8.85)
Follower Trait Anxiety	0.82***	(42.00)	0.82***	(41.65)	0.82***	(41.71)
Leader Trait Anxiety			0.17	(1.69)	0.01	(0.06)
Leader State Anxiety (Day 1) X Leader Trait Anxiety			-0.10*	(-2.08)	-0.02	(-0.34)
Corona					-0.88*	(-2.07)
Corona X Leader State Anxiety (Day 1)					0.42*	(2.13)
Corona X Leader Trait Anxiety					0.42*	(2.10)
Corona X Leader State Anxiety (Day 1) X Leader Trait Anxiety					-0.19*	(-2.10)
Constant	0.21**	(2.89)	-0.20	(-0.86)	0.15	(0.53)
AIC	7240.48		6925.35		6903.11	
BIC	7492.88		7194.29		7206.75	

Note: All models include additional controls, namely organization size (i.e., number of employees; categorical variable), leader and follower Big Five personality traits, leader and follower gender (0 = female, 1 = male) and time-effects; COVID-19 coded 0 (5^th^ October 2019 – 10^th^ March 2020) and 1 (11th March 2020 – 13^th^ August 2020); z-statistics in parentheses

* *p* < .05

** *p* < .01

*** *p* < .001, n = 44,506 daily dyads (197 leaders and 958 followers).

All outlined models in Table 2 include various control variables, as noted below in the respective table. To minimize the potential of an unaccounted “third variable” [14] causing a shift in both leader and follower state anxiety on the same day, we apply a multi-day lagged regression design. Doing so allows us to test the following: if a leader posts a highly (or less) anxious tweet today, are respective followers more likely to post (less) anxious tweets on subsequent days? The proposed lag analysis also allowed us to account for potential sleeper effects of the leader-follower anxiety influence.

In Table 2 we present the results of this multi-day lagged regression design, namely by examining whether leader state anxiety on Day 1 predicts follower state anxiety on Day 2, controlling for both leader and follower state anxiety (Day 0 and Day 1), respectively. We also controlled for possible spillover effects of follower state anxiety on preceding days (i.e., Day 0 and Day 1) and of leader state anxiety on preceding days (i.e., Day 0). The main two-way interaction was significant (Table 2, M2: *b* = 0.10, *SE* = 0.05, *z* = -2.08, *p* = 0.037).

### Context as a moderating factor

**Fig 1:**
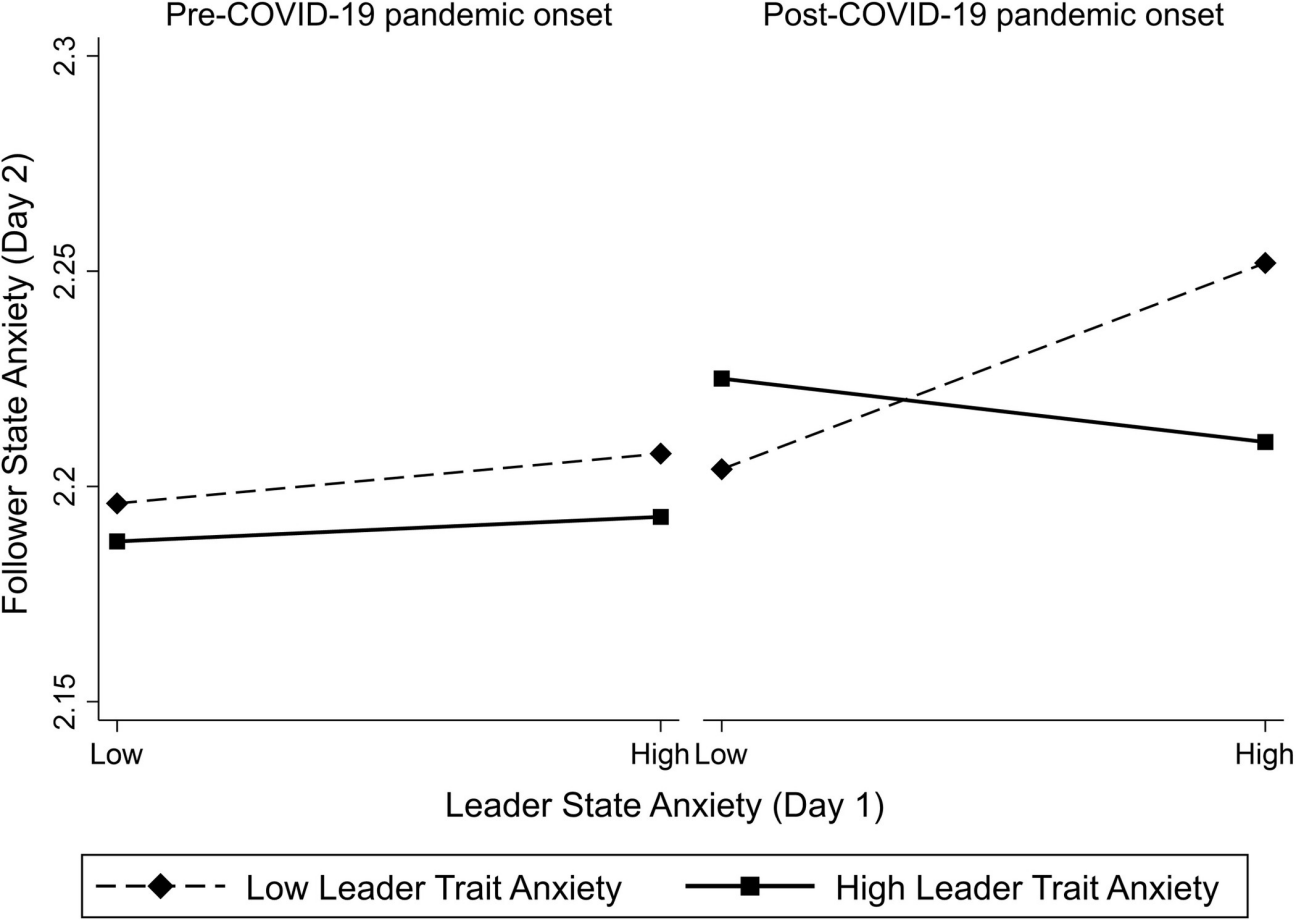
Regression interaction between leader state- and leader trait anxiety on follower state anxiety.

We further examine our findings in the context of the COVID-19 pandemic. To do so, we created a dummy variable, which specifies the pre-and-post onset of COVID-19 (11^th^ March 2020, the WHO declares COVID-19 a worldwide pandemic). Model 3 (Table 2) includes the examined three-way interaction and all considered controls (identical to M1 and M2, Table 2). We find a significant three-way interaction, between leader state- and trait-anxiety on follower anxiety and the pre-and-post onset of the COVID-19 pandemic(Table 2, M3: *b* = -0.19, *SE* = .09, *z* = -2.10, *p* = 0.036). To better understand this interaction, we plotted the results of the complete model (Table 2, M3) in Fig 1.

Graphing this three-way interaction (5^th^ and 95^th^ percentile, Fig 1) showed that in the case of less trait anxious leaders, leader state anxiety on Day 1 was associated positively with high follower state anxiety on the next day during the pandemic (simple slope = 0.06, *SE* = 0.02, *z* = 3.53, *p* < .001). Hence, it seems that in a crisis context, followers of less trait anxious leaders seemed to be most impacted by increased leader state anxiety than followers of more trait anxious leaders.

In the case of trait anxious leaders, this trend was not significant (simple slope = -0.02, *SE* = 0.01, *z* = -1.26, *p* > 0.10). Results before the onset of the COVID-19 pandemic were even less pronounced and proved to be not significant both in the case of less trait anxious (simple slope = 0.01, *SE* = 0.01, *z* = 1.03, *p* > 0.10) as well as trait anxious leaders (simple slope = 0.01, *SE* = 0.01, *z* = 0.60, *p* > 0.10).

#### Robustness checks

We also tested the same three-way interaction using a continuous-time variable instead of the aforementioned dummy variable. Results remained unchanged with the examined three-way interaction between leader state anxiety (Day 1) predicting follower state anxiety (Day 2) over time (*b* = -.00, *SE* = 0.00, *z* = -2.29, *p* = 0.02).

## Discussion

Previous research has found that leaders effectively transfer emotions to their followers in face-to-face communications. However, less clarity exists concerning the transfer of emotion via CMC in a naturally occurring environment.

In this study, we find that leader state anxiety predicts follower state anxiety, even when accounting for a series of leader and follower personality traits and demographics (e.g., leader and follower trait anxiety, gender, etc.). We also find that follower state anxiety is a function of both leader state- (i.e., leaders’ experience of event-specific anxiety over time) and leader trait anxiety (i.e., leaders’ tendency to experience anxiety in general). Hence, followers of highly trait anxious leaders and who are experiencing increased state anxiety (e.g., due to COVID-19 pandemic and its repercussions), experience less state anxiety than followers of less trait anxious leaders. Put differently, followers of leaders who do not tend to be anxious in general (unrelated to a specific event), seem to be less used to their leaders’ affect and might be more susceptible to their leaders’ increased state anxiety. This could be because followers of trait anxious leaders might discount their leaders’ expressed anxiety and instead look for emotional cues from other important people in their lives (e.g., peers, colleagues, etc.), while the same is not the case for followers of less trait anxious leaders. Relational individual differences might play a role here as well [e.g., 42].

## Implications

We argue that the study of anxiety as a follower outcome is particularly important because the experience of anxiety can have destructive consequences [19], including depleted self-regulation, and increased and prolonged emotional exhaustion [6]. In addition, anxious individuals, in their aim to lower their anxiety oftentimes reach out to others for advice, but also are more likely to accept un-advantageous or even inappropriate and harmful advice from others [43]. Finally, the transfer of anxiety from leader to follower is important because it can serve as a precursor of the subsequent cascading emotional contagion between team members, which can determine team affect over time. Hence, we suggest that even low rates of “anxiety spread” may be meaningful in influencing team members’ affect over time.

We found that the proposed effects seem to be dependent on the contextual effects of the COVID-19 pandemic. The COVID-19 crisis emerged very fast with an all-encompassing impact on individuals, organizations, and communities. Although there are similarities between the COVID-19 pandemic and previous crises regarding economic consequences, the COVID-19 crisis brings to the forefront critical health and safety issues as well. For example, social distancing created serious knock-on effects on the way people communicate, collaborate, and work [44]. This in turn means that the use of social platforms of communication increased dramatically with various consequences including mental health. In our study, we found that the transfer of anxiety via social media from leaders to followers is stronger throughout the pandemic compared to the examined pre-pandemic period. In that sense, we suggest that the role of crisis context is of particular importance in transferring anxiety between leaders and followers and should not be ignored. The crisis context makes people more vulnerable and stressed resulting in decreasing their commitment to organizational leaders and their organization overall [45]. In this respect, leaders need to understand that during a crisis everything counts, even the words they are publicly expressing via social media. There is a specific trend for leaders to try to take more responsibility and respond to employees’ demands during a crisis period [46]. However, this seems not to be always the ideal action, since some evidence suggests that leaders’ effort to take responsibility during a crisis and communicate their thoughts can harm their own [47] as well as their followers’ well-being [48]. This also seems to be the case when communicating online based on the results of this study. Since social media is becoming a dominant way of triggering collective behavior in organizations [49]; leaders that once used to influence their follower’s anxiety levels in face-to-face communication, seem to equally affect their emotional status by simply tweeting. In this respect, our study demonstrates the potential negative implications of leadership communication via social media by highlighting the impact of being a communicative leader under adverse situations on follower well-being. Based on our findings, one clear implication is related to the way that leaders can use social media communication. We suggest that leaders should be more aware of the impact of their communication to others using CMC and learn to behave more strategically, specifically avoiding comments on issues that might be be perceived as negative by their employees. In addition, mentoring programs or even CMC training could potentially help leaders develop their social media communication skills.

## Limitations and future research

Our study is not without limitations. Firstly, although our applied ML methodology allows us to score millions of tweets across a long period of time, it could be argued that social media profiles might not reflect actual personality but rather an idealized form of user representation. Yet, previous research has found strong support for assessing personality traits in social media posts [38, 39]. Indeed, we would argue that the anxiety detection algorithm specifically is superior to self-report ratings of anxiety because the onset of anxiety is not always immediately evident to the experiencing person and lead to an increased likelihood of burnout and exhaustion over time if not recognized. However, due to the behavioral residue in CMC, more accurate changes in anxiety can be detected [31].

Secondly, one could argue that the leader-follower dyads in the present paper are not sufficient, since we do not guarantee that followers actually read their leaders’ posts. Based on the work of Kramer [14], we argue that it would not be computationally feasible to assess whether followers read all of their leaders’ posts. For example, a follower viewing their leaders’ newsfeed likely will be presented with the most recent or important (i.e., pinned) posts, with more posts appearing on each page-down, which go unread. It would be unlikely to expect that social media users’ affect is influenced by unread posts. Hence, our decision to not systematically include or exclude followers based on which leader posts followers had viewed constitutes an error, which would only make it more difficult to find statistically significant results [14]. Yet, we do find that leaders’ tweets are significantly associated with follower state anxiety as expressed in followers’ subsequent posts.

Thirdly, we recognize that we could have restricted our data collection approach to followers who actively commented on their leaders’ posts. While this might have increased our observed effect size, we chose not to restrict our sample in this way, due to possible existing disclosure norms [50]. We argue that it is likely that even if the transferred leader emotion is not felt by their followers, followers might feel required to respond, most likely in agreement with and in support of their leaders’ respective post content and tone. This is likely because a) organizational leaders hold positions of status, authority, and power over lower-ranked employees [4] and b) interactions on Twitter constitute public statements. Moreover, leaders who communicate using social media usually do not direct their posts to specific followers but rather use these platforms to communicate with entire communities. Hence, by not restricting our sample to only responding followers, we can be confident that any observed effects are not due to disclosure norms or proximal communication. Finally, although the presented regression models are based on multi-day lagged analyses, in which a variable on a given day predicts another variable on subsequent days, this study is not based on an experimental research design and therefore causal claims cannot be made to the same extent. However, we would argue that studying wide-reaching phenomena such as the effect of a real (not simulated) crisis context in such a large sample would be difficult if not impossible to do using a truly experimental research design, as randomizing participants into various experimental conditions is likely not feasible and brings with ethical considerations as well. Instead, the presented approach allows the study of interactions in a naturally occurring non-obtrusive manner.
